# Layered Hybrid Perovskites for Highly Efficient Three‐Photon Absorbers: Theory and Experimental Observation

**DOI:** 10.1002/advs.201801626

**Published:** 2018-12-20

**Authors:** Shunbin Lu, Feng Zhou, Qi Zhang, Goki Eda, Wei Ji

**Affiliations:** ^1^ SZU‐NUS Collaborative Innovation Centre for Optoelectronic Science and Technology International Collaborative Laboratory of 2D Materials for Optoelectronic Science and Technology of Ministry of Education College of Optoelectronic Engineering Shenzhen University Shenzhen Guangdong 518060 P. R. China; ^2^ Department of Physics National University of Singapore Singapore 117551 Singapore; ^3^ Department of Chemistry National University of Singapore Singapore 117542 Singapore; ^4^ Centre for Advanced 2D Materials National University of Singapore Science Drive 2 Singapore 117546 Singapore

**Keywords:** 2D excitons, hybrid perovskites, nonlinear optics, three‐photon absorbers

## Abstract

Multiphoton absorption may find many technological applications, such as enhancing the conversion efficiency of solar cells by the utilization of sub‐band‐energy photons, below‐bandgap photodetection through the simultaneous absorption of several infrared photons for photocurrent generation, or light frequency upconversion for high‐resolution, 3D imaging. To enhance multiphoton absorption in semiconducting materials, one of the strategies is to explore low‐dimensional excitons. Here, a quantum perturbation theory on a giant enhancement in three‐photon absorption (3PA) arising from 2D excitons in multilayered crystals of organic–inorganic hybrid perovskites is presented. The maximal 3PA coefficient is predicted to be in the range of 2–7 cm^3^ GW^−2^ at 1100 nm, the largest values reported so far for any 2D and bulk semiconductors at room temperature. Excellent agreement between theory and the experimental findings unambiguously demonstrates a pivotal role in the enhancement of 3PA played by 2D excitons. The theory predicts that the resonant 3PA coefficient should be enhanced further by at least two orders of magnitude with very low temperature. The findings are essential for understanding giant 3PA arising from 2D excitons in layered hybrid perovskites and may open new pathways for highly efficient conversion from infrared light energy to either electrical energy or higher‐frequency light emission/lasing.

## 1 Introduction

Organic–inorganic hybrid perovskites have recently emerged as a class of promising materials for a variety of optoelectronic and photonic applications, among which, both converting solar energy to electrical energy^1^, ^2^ and multiphoton‐pumped lasing^3^, ^4^ have received increasing attention. In order to further enhance the solar‐to‐electrical conversion efficiency, one of the reported strategies is to explore multiphoton absorption (MPA) processes which can convert the sub‐band photon energy into the electrical energy.^5^ In multiphoton‐pumped lasing technologies, MPA is required to create population inversion in a lasing material. In general, however, high light irradiance levels are required for such multiphoton processes. Therefore, many research works have been carried out toward the considerable reduction in light irradiances required for MPA.

On one hand, there have been many experimental reports^5^, ^6^, ^7^, ^8^, ^9^, ^10^, ^11^, ^12^, ^13^, ^14^, ^15^ on the enhancement of MPA coefficients or cross‐sections if one utilizes nanostructured perovskites, such as in 2D forms, or quantum dots (QDs). For example, the three‐photon absorption (3PA) action cross sections have been reported as high as 10^−73^ cm^6^ s^2^ photon^−2^ for perovskite QDs with diameters of 8–10 nm.^15^ On the other hand, however, there is no literature report on the theoretical explanations why nanostructured perovskites exhibit such giant MPA properties. Previously, we have developed theoretical models to quantitatively explain large MPA in monolayer transition metal dichalcogenides.^16^, ^17^, ^18^ By applying a quantum perturbation theory to 2D excitons in monolayer transition metal dichalcogenides, our models predict the spectra of two‐photon absorption (2PA) and 3PA coefficients, which are several orders of magnitude higher than their counterparts in bulk crystals based on the two‐band models. Such a huge enhancement is attributed to the 2D nature of excitons; and in particular, larger electric dipole moments for electronic transitions among highly energetic excitonic states (or Rydberg states) make noticeable contributions to the enhancement. Our theoretical results have also been confirmed by experimental measurements in monolayer transition metal dichalcogenides.^16^, ^17^, ^18^

In order to demonstrate that our theory should be valid to any 2D semiconducting crystals, here, we extend our 2D excitonic quantum perturbation theory to 2D perovskites, and we report a significant enhancement in the 3PA coefficients of 2D perovskites as compared to 3D perovskites, by both theoretical prediction and experimental observation. Our findings open a promising avenue for a significant decrease of the required light irradiances for MPA in the infrared regime, leading 2D perovskites to an advantage in many photonic applications, like multiphoton‐pumped lasing, light frequency upconversion, 3D biomedical imaging, and harvesting solar energy.

**Figure 1:**
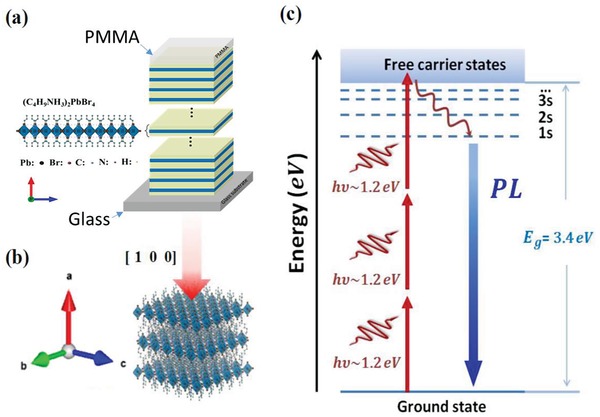
Crystal configurations and energy diagram: a) A schematic of layered perovskites on a glass substrate, and the layered perovskites are covered by PMMA. b) A schematic on the structure of trilayer (C_4_H_9_NH_3_)_2_PbBr_4_, which is excited by linearly polarized light (red arrow) at normal incidence of [1 0 0] plane. c) Energy level diagram for three‐photon absorption (3PA) and photoluminescence (PL) in the layered perovskite crystal. The dashed lines are the energy levels of s‐excitons. The red and blue arrows show 3PA and subsequent PL processes, respectively. The wiggle arrow shows the relaxation transition from free carrier states (or conduction band electrons) to an excitonic state.

**Figure 2:**
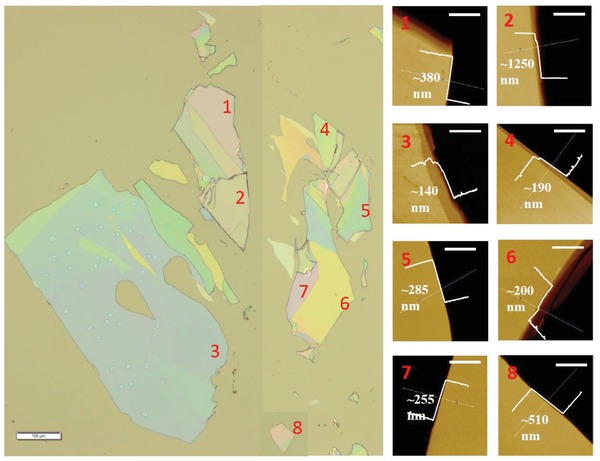
Lateral shapes and thicknesses. The left part: A CCD photo image of 2D hybrid perovskite samples. The numbers mark the lateral positions where 3PA‐excited photoluminescence (3PPL) was investigated. Scale bar: 100 µm. The right part: eight atomic force microscopic (AFM) images showing the thicknesses of corresponding eight positions in the CCD photo (left). Scale bar: 3 µm.

**Figure 3:**
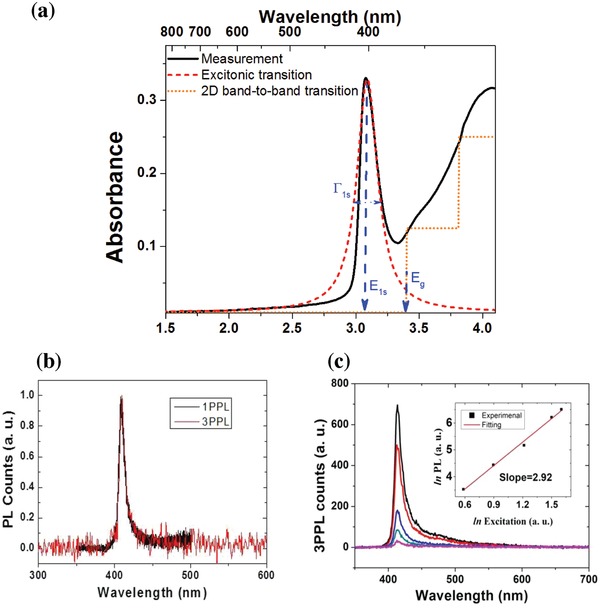
a) One‐photon absorption spectrum (black) on the samples shown in the left part of Figure 2. The red curve is the best fit by a Lorentzian lineshape function, from which the values of *E*_1s_ and Γ_1s_ are extracted. The dotted orange line is a step function to illustrate the band‐to‐band absorption in 2D crystals. b) Measured spectra for both one‐photon‐excited and three‐photon‐excited photoluminescence (1PPL and 3PPL). Thickness of multilayered perovskite (C_4_H_9_NH_3_)_2_PbBr_4_: 140 nm. c) 3PPL spectra recorded at various excitation peak irradiances. The inset shows the 3PPL versus the excitation irradiance on the log–log scale. Excitation wavelength: 1030 nm, and thickness of multilayered perovskite (C_4_H_9_NH_3_)_2_PbBr_4_: 380 nm.

**Figure** **1** illustrates a 2D perovskite system that renders large 3PA at laser excitation of 1030 nm wavelength (or 1.2 eV photon energy). Each layer of perovskite ((C_4_H_9_NH_3_)_2_PbBr_4_) is composed of one inorganic slab (PbBr_4_^2−^) and two organic layers (C_4_H_9_NH_3_^+^). Such an organic–inorganic 2D system was chosen because of the recent report for its high photoluminescence (PL) efficiency.^1^ We have fabricated such 2D systems with total layer numbers between 100 and 910 (or total crystal thicknesses between 140 and 1250 nm), see **Figure** **2**, and the fabrication details can be found in the Experimental Section. Light absorption results in the generation of electron–hole pairs, leading to excitons (or hydrogen‐like atoms) due to the Coulomb force. **Figure** **3**a presents an experimentally observed absorption spectrum, supporting that such excitons manifest themselves pronouncedly at room temperature. Because of the two organic layers acting as barriers, such excitons are confined within a 2D space, and hence, their energy levels or wave functions are described by the 2D hydrogen‐like atomic model, which is quantitatively different from its 3D counterpart.

Based on the 2D excitonic model with the electric dipole approximation and the energy diagram in Figure 1c, the degenerate 3PA coefficient, α_3_, can be approximated as a function of photon energy, *hv*, by^16^

(1) $$\begin{array}{ll} \alpha_{3}\left(h\nu \right) & =CN{\left(h\nu \right)}^{3}{\left[\frac{E_{\mathrm{loc}}}{E}\right]}^{6}\frac{{\left|\mu_{\mathrm{sg}}\right|}^{2}}{{\left(E_{1s}-h\nu \right)}^{2}+{\left(\Gamma_{1s}/2\right)}^{2}}\\ & \;\times \left[\sum_{p,f}\frac{{\left|\mu_{\mathrm{ps}}\right|}^{2}}{{\left(E_{\mathrm{pg}}-2h\nu \right)}^{2}+{\left(\Gamma_{p}/2\right)}^{2}}\frac{{\left|\mu_{\mathrm{fp}}\right|}^{2}\Gamma_{f}/2\pi }{{\left(E_{\mathrm{fg}}-3h\nu \right)}^{2}+{\left(\Gamma_{f}/2\right)}^{2}}\right] \end{array}$$

where *C* is a material‐independent constant which has a value of 1.0 × 10^76^ in the units such that α_3_ is in cm^3^ GW^−2^; *N* is the density of active unit cells per cm^3^; *hv*, *E_ij_*, and Γ_*i*_ are the photon energy, the energy difference between two energy levels: *i* and *j*, and the linewidth, respectively, in units of eV; μ_*ij*_ is the electric dipole moment between the two energy levels in units of esu; $\frac{E_{\mathrm{loc}}}{E}\;=\;\frac{1}{3}\;(n_{0}{}^{2}+1)$ is the local‐field correction with *n*_0_ being the refractive index; and *E*_1s_ and Γ_1s_ are the lowest s‐exciton energy and the linewidth, respectively.

In order to increase the 3PA coefficient, as shown by Equation (1), one may take advantage of a solid material with a large refractive index or dielectric constant, as a recent demonstration for a significant enhancement in MPA.^19^ Another way to increase α_3_, one may explore the electric dipole moment. For the 3PA, there are three electric dipole moments involved, corresponding to three electronic transitions. In Equation (1), μ_sg_ corresponds to the 1s‐exciton generation by absorbing a photon. As for the second dipole moment, μ_ps_, only transitions between 1s‐excitons and 2p‐ or 3p‐excitons are taken into consideration in our model. And the third dipole moment, μ_fp_ corresponds to the transition between one of 2p‐ and 3p‐excitons and one of s‐ and d‐excitons with the primary quantum number greater than 3. Because these highly energetic states are partially overlapping within the linewidths, a summation (Σ_p,f_) is required to account for all their contributions to the 3PA. Moreover, only one conduction band and one valence band involved, Equation (1) is simpler than the case where one conduction and two valence bands are taken into consideration for monolayer MoS_2_.^16^

The electric dipole moment, $\mu_{ij}=〈\psi_{j}|e\;\vec{r}\cdot \vec{u}|\psi_{i}〉$ where $\vec{u}\;$ is the unit vector for the electric field direction of light, is proportional to the integral over the product of the two wave functions: one for the lower energy state, ψ_*i*_ and the other for the upper energy state, ψ_*j*_. To compare the difference between the 2D and 3D excitons, we assume the wave functions of hydrogen atoms in the dipole moment calculation, and they are given by^20^, ^21^

(2) $$|\psi_{n,m}\left(r\right)〉=\frac{1}{a_{B}^{2D}}\sqrt{\frac{q_{0}{}^{3}\left(n-\left|m\right|-1\right)!}{\pi \left(n+\left|m\right|-1\right)!}}{\left(\frac{2q_{0}r}{a_{B}^{2D}}\right)}^{\left|m\right|}e^{-\frac{q_{0}r}{a_{B}^{2D}}}L_{n-\left|m\right|-1}^{2\left|m\right|}\left(\frac{2q_{0}r}{a_{B}^{2D}}\right)e^{-im\varphi }$$

and

(3) $$\begin{array}{ll} |\psi_{n,l,m}\left(r\right)〉 & =\sqrt{{\left(\frac{2}{na_{B}^{3D}}\right)}^{3}\frac{\left(n-l-1\right)!}{2n{\left[\left(n+l\right)!\right]}^{3}}}{\left(\frac{2r}{na_{B}^{3D}}\right)}^{l}e^{-\frac{r}{na_{B}^{3D}}}\\ & L_{n-l-1}^{2l+1}\left(\frac{2r}{na_{B}^{3D}}\right)Y_{l}^{m}\left(\theta ,\varphi \right) \end{array}$$

where $q_{0}=\frac{1}{n-1/2}\;,$ *n*, *l*, and *m* are the principle quantum number, the angular momentum quantum number, and the magnetic quantum number, respectively; $a_{B}^{2D}$ and $a_{B}^{3D}\;$ correspond to the effective Bohr radius in the 2D and 3D case, respectively; *L*(*x*) is the generalized Laguerre polynomial; ϕ is the rotational angle in the orbital; and *Y*(*x*) is the spherical harmonics.

## 2 Results and Discussion

**Figure 4:**
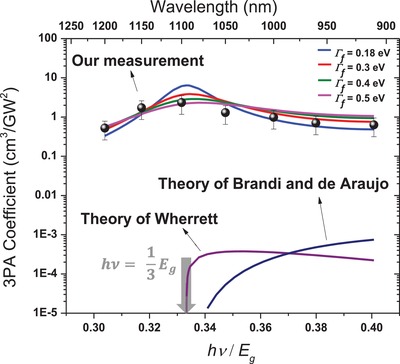
Experimentally measured (black circles) and theoretically calculated (colored curves) 3PA spectra. Theories are calculated by Equation (1).^22^, ^23^ Γ_f_ is the linewidth of the final state for 3PA transitions, and *E*_g_ = 3.4 eV.

All the parameters in Equation (1) can be experimentally determined, except for Γ_f_, the linewidth of the final state for the 3PA transition. In our calculations, we treat this linewidth as an adjustable parameter. By taking experimentally determined values in Table S1 (Supporting Information) with Γ_f_ values being in the range from 0.18 to 0.5 eV, the 3PA coefficients are calculated for the near‐infrared spectral region and are shown by the colored curves in **Figure** **4**.

**Table** **1** shows the calculated electric dipole moments for transitions from 1s‐ to np‐excitons. Obviously, the dipole moments are less important when *n* > 3, and hence they are neglected justifiably in our model. The enhancement in 3PA by 2D excitons results from the two transitions, that is, from 1s‐ to 2p‐(or 3p‐) exciton; and from 2p‐(or 3p‐) state to highly excited Rydberg states. In **Table** **2**, the comparison between the 2D and 3D exciton is shown, and the ratio of $|\mu_{\left(\mathrm{np})(1s\right)}^{2D}\mu_{\left(n\prime s)(\mathrm{np}\right)}^{2D}{|}^{2}$ to $|\mu_{\left(\mathrm{np})(1s\right)}^{3D}\mu_{\left(n\prime s)(\mathrm{np}\right)}^{3D}{|}^{2}$ increases dramatically as *n*′ increases, where *n*′ > 3.

**Table 1:** Excitonic energy levels and square of 1s‐to‐np electric dipole moments

*n*	$E_{\mathrm{np}}^{\mathrm{2D}}\;\;=\left(E_{g}-\frac{0.0825}{{\left(n-1/2\right)}^{2}}\right)\;\;\mathrm{eV}$	$|〈\;\varphi_{\mathrm{np}}^{\mathrm{2D}}|e\;\vec{r}\cdot \vec{u}|\varphi_{\mathrm{1s}}^{\mathrm{2D}}\;〉{|}^{2}\times \;{\left(10^{-18}\;\mathrm{esu}\right)}^{2}$
2	3.363	41.4
3	3.387	6.9
4	3.393	2.6
5	3.396	1.1

**Table 2:** Squares of electric dipole moments and their ratios

Transitions	${\left|\mu_{\left(\mathrm{np}\right)\left(1s\right)}^{2D}\;\mu_{\left(n^{\prime }s\right)\left(\mathrm{np}\right)}^{2D}\right|}^{2}\times \;\;{\left(10^{-18}\;\mathrm{esu}\right)}^{4},\;\left(n^{\prime }\;>n\right)$	$\frac{{\left|\mu_{\left(\mathrm{np}\right)\;\left(\mathrm{1s}\right)}^{\mathrm{2D}}\;\mu_{\;\left(n^{\prime }s\right)\;\left(\mathrm{np}\right)}^{\mathrm{2D}}\right|}^{2}}{{\left|\mu_{\left(\mathrm{np}\right)\;\left(\mathrm{1s}\right)\;}^{\mathrm{3D}}\mu_{\;\left(n^{\prime }s\right)\;\left(\mathrm{np}\right)}^{\mathrm{3D}}\right|}^{2}}$
1s→2p→3s	8.17 × 10^3^	2.2 × 10^1^
1s→2p→s	1.48 × 10^3^	4.1 × 10^2^
1s→2p→s	5.42 × 10^2^	1.1 × 10^4^
……		
1s→p→s	1.11 × 10^4^	2.0 × 10^4^
1s→p→s	1.88 × 10^3^	2.3 × 10^5^
1s→p→s	6.76 × 10^2^	8.8 × 10^6^
……		

To validate the above theoretical model, we measured the 3PA‐excited photoluminescence (3PPL) from our 2D perovskite samples. These measurements were conducted with two configurations of experimental setup. In the first setup, the samples were examined in detail under a confocal microscope. Their lateral shapes and thicknesses are displayed in Figure 2. The thicknesses were varied from 140 to 1250 nm. By the excitation of 1030 nm (or ≈1.2 eV) femtosecond laser pulses with 1 MHz repetition rate, the measured 3PPL shows the same spectral profiles as those excited by one‐photon absorption, see Figure 3b as example. In addition, it also exhibits a nearly cubic dependence on the excitation irradiance, see an example in Figure 3c. (More measurements including thickness dependence, excitation polarization dependence, and photostability can be found in the Supporting Information.) In the second setup, femtosecond laser pulses with 1 kHz repetition rate and wavelengths in the range from 900 to 1200 nm were employed as an excitation source to excite the samples shown in the left part of Figure 2. The measured wavelength‐dependent 3PPL spectra are shown in Figure S7 (Supporting Information), from which the 3PA coefficients were extracted and are plotted versus wavelength in Figure 4.

By comparison, in Figure 4, we also present two theories developed by Brandi and de Araujo,^22^ and Wherrett^23^ for 3PA transitions only taking placing between the conduction and valence band in a corresponding bulk crystal with the same bandgap, effective masses, and refractive index. Figure 4 clearly shows that the 3PA coefficients arising from electronic transitions among the 2D excitonic states are three orders of magnitudes higher than their counterparts resulting from the band‐to‐band transitions at *hv*/*E*_g_ ≈ 0.37, where *E*_g_ is the bandgap.

Depending on the value of the linewidth, Γ_f_, the maximal 3PA coefficient is in the range from 2 to 7 cm^3^ GW^−2^ at ≈1100 nm. It is three orders of magnitude greater than the experimental measured value (6 × 10^−3^ cm^3^ GW^−2^) for ZnSe at a wavelength of 1310 nm.^24^ Compared to the measured 3PA coefficient of Si^25^ in the spectral range of 2.3–3.3 µm, the values of 2–7 cm^3^ GW^−2^ are also larger by three orders of magnitude as well. Furthermore, the maximal 3PA for direct band transitions in Ge has been measured to be 0.27 cm^3^ GW^−2^ at 4 µm,^26^ one order of magnitude less than the values presented in Figure 4 at ≈1100 nm. The 3PA coefficient was measured to be 0.05 cm^3^ GW^−2^ at 1064 nm for a bulk crystal of MAPbCl_3_ perovskite,^27^ which is also significantly less than our theoretical results at 1100 nm.

By utilizing the same 2D excitonic model, we find that the maximal 3PA coefficient of 2D perovskites is at least one order of magnitude greater than the maximal 3PA coefficient of monolayer MoS_2_.^16^ This is mainly due to a larger refractive index (*n*_0_ = 2.5), as compared to *n*_0_ = 1.84 in monolayer MoS_2_. In addition, our experimental results demonstrate that the interaction length can be scaled up to 300 nm, see Figure S5 (Supporting Information). In the thickness range up to 300 nm, the measured 3PPL is approximately proportional to the thickness. It is expected as the excited volume is proportional to the thickness. Beyond 300 nm, the measured 3PPL becomes saturated with the thickness, due to the fact that the one‐photon absorption and 3PPL is partially overlapping as shown in Figure 3b and Figure S4 (Supporting Information), and hence, the reabsorption of 3PPL becomes significant as the thickness is beyond 300 nm.

As for polarization‐dependent 3PPL measurements, we define θ as the angle between the light polarization vector and the crystallographic axis when the propagation direction is along the [1 0 0] direction, see Figure S6 (Supporting Information). The measured polarization dependence for C_8_H_24_N_2_PbBr_4_ can be fitted by using *y* = *A*{1 + 2[σ_1_sin^4^(θ + ϕ) − σ_2_sin^2^(θ + ϕ)]} with σ_1_ = −2.67 and σ_2_ = −2.89 and parameters *A*, σ_1_, σ_2_, and ϕ are defined in ref. 27, indicating a significant anisotropy in χ^(5)^ at room temperature. It is also in agreement with the tetragonal structure of C_8_H_24_N_2_PbBr_4_ perovskites in plane. Furthermore, with α_3_ = 7 cm^3^ GW^−2^ and peak irradiance *I*_00_ = 40 GW cm^−2^, we estimate that exciton density is ≈2 × 10^11^ cm^−2^, at which other excitonic effects (such as bi‐excitons, tri‐excitons) are not so important.

It was reported that the 3PA coefficient is as large as 10 cm^−1^ (MW cm^−2^)^−2^ = 10^7^ cm^3^ GW^−2^ for hydrogen‐like donors in silicon in the THz regime at 10 K.^19^ Such an enormous 3PA coefficient is partially due to a hydrogenic oscillator scale being inversely proportional to ν^3^.^19^ By considering the two‐orders‐of‐magnitude difference between infrared and THz wave frequencies, the 3PA coefficient in the infrared is anticipated to be reduced by six orders of magnitude. Furthermore, the giant α_3_‐value was observed when the linewidth of the excited state was very narrow, ≈50 GHz corresponding to 2.1 × 10^−4^ eV, obtained at 10 K. Our model depends on the linewidth which, in turn, is related to the sample temperature. Figure S1 (Supporting Information) shows the numerical modeling, supporting that the resonant 3PA coefficient is enhanced by two orders of magnitude if the linewidth is decreased from 0.1 to 0.001 eV. The empirical fitting to the numerical modeling reveals that α_3_ ∝ Γ_f_^−0.9^. Although our 3PA coefficients in 2D perovskites are significantly less than hydrogen‐like donors in silicon at 10 K, our values are the largest in the infrared spectral region at room temperature, which are of direct relevance to photonic applications including both optical communications and conversion of sub‐band solar energy to electrical energy.

## 3 Conclusion

In summary, we have presented a quantum perturbation theory on a giant enhancement in 3PA arising from 2D excitons in multilayered crystals of organic–inorganic hybrid perovskites, as compared to bulk crystals of perovskites. The maximal 3PA coefficient in 2D perovskites is predicted to be 2–7 cm^3^ GW^−2^, the largest values reported so far for any 2D and bulk crystals in the near‐infrared spectral region at room temperature. Excellent agreement between our theory and our experimental findings unambiguously demonstrate a pivotal role in the enhancement of 3PA played by 2D excitons. Our theory predicts that the resonant 3PA coefficient should be enhanced further by at least two orders of magnitude if the temperature is low such that the linewidth of the final excitonic state is below 1 meV. Our findings are essential for understanding giant 3PA arising from 2D excitons in layered perovskites and may open new pathways for highly efficient conversion from infrared light energy to either electrical energy or higher‐frequency light emission/lasing.

## 4 Experimental Section

*Chemical Materials*: Butylamine (≥99%), N,N‐dimethylformamide (ACS reagent, ≥99.8%), dichloromethane (ACS reagent, 99.5%), hydrobromic acid (ACS reagent, 48%), and lead (II) bromide (≥98%) were used. All chemicals were purchased and used without further purification.

*Synthesis of (C_4_H_9_NH_3_)_2_PbBr_4_ Bulk Crystal*: Single‐crystalline (C_4_H_9_NH_3_)_2_PbBr_4_ was synthesized by antisolvent vapor assisted crystallization process. Basically, stoichiometric amount of ammonium salt (C_4_H_9_NH_3_Br) and lead bromide (PbBr_2_) was dissolved in N,N‐dimethylmethanamide (DMF) to form 0.5 m precursor solution and it was sealed in a beaker surrounded by dichloromethane (CH_2_Cl_2_) working as an antisolvent. Plate‐like (C_4_H_9_NH_3_)_2_PbBr_4_ crystal precipitates from precursor solution since antisolvent vapor lowers down the solubility of hybrid perovskite precursors. Bulk crystal was obtained by suction filtration and then stored in a glove box for later usage.

*X‐Ray Diffraction Characterization*: Single‐crystal X‐ray diffraction to verify the crystal structure was performed on Bruker AXS D8 Venture which was equipped with a Photon 100 CMOS active pixel sensor detector. A molybdenum monochromatized (λ = 0.71073 Å) X‐ray radiation was used. Data reduction and numerical absorption corrections were performed using the APEX3 suite. All structures were solved by direct methods and refined by full‐matrix least‐squares on F^2^ using the Bruker SHELXTL software package. The crystal is orthorhombic, space group Cmca. The asymmetric unit contains half a molecule of C_8_H_24_N_2_PbBr_4_. The cation was disordered into two positions with occupancy ratio = 50:50. Restraints in bond lengths and thermal parameters were applied to the disordered atoms. Final *R*‐values are *R*_1_ = 0.0655 and *wR*_2_ = 0.1673 for 2θ up to 56°. The results are summarized in Table S2 (Supporting Information).

*Sample Fabrication*: Layered (C_4_H_9_NH_3_)_2_PbBr_4_ flakes were mechanically exfoliated from the above‐described bulk crystal, and transferred to a precleaned glass substrate in the cleanroom where then they were also characterized by both Raman spectroscopy (WITec ALPHA 300R), see Figure S2 (Supporting Information) and atomic force microscopy (Bruker, Dimension FastScan) in air ambient environment. Finally, perovskite samples were encapsulated with poly(methyl methacrylate) (PMMA) thin film (A7, 950) by spin‐coating (SPS SPIN 150, 2000 rpm, 60 s) and annealing on a hotplate (SD162) at 90 °C for 10 min.

*One‐Photon Absorption and One‐Photon‐Excited Photoluminescence (1PPL)*: A confocal microscope was utilized. Its setup is schematically shown in Figure S3 (Supporting Information). For one‐photon absorption measurements, white light from a light source (Spectra Products, Xenon Light Source, ASB‐XE‐175) was illuminated on the perovskite sample and its transmitted light was channeled to a spectrometer (Ocean Optics QEpro). To measure 1PPL, a CW He‐Cd laser (excitation wavelength: 325 nm) was focused by an objective lens through the glass substrate onto the selected area (≈3 µm in diameter) of the perovskite sample and 1PPL was collected by the objective lens and channeled to the spectrometer.

*Three‐Photon‐Excited Photoluminescence*: The same confocal microscope (Figure S3, Supporting Information) was employed with a femtosecond pulsed laser (Light Conversion Pharos‐9W fs oscillator with wavelength: 1030 nm; repetition rate: 1 MHz; and pulse duration (HWe^−1^M): ≈110 fs and polarization: linear). The orientation of the linear polarized laser pulses was varied by a half wavelength plate. The laser pulses were focused by the objective lens through the glass substrate onto the selected area (≈6 µm in diameter) of the perovskite sample. To obtain the 3PA action coefficient (*ηα*_3_) of the perovskite sample, the second harmonic generation (SHG) signal from a BBO crystal was used under the same setup as a reference. The reflected SHG signal by the top surface of the BBO crystal was collected by the same objective lens and was channeled to the spectrometer. For the SHG operated in the tightly focused regime, the analytical analysis can be found in ref. 28. The ratio of the 3PPL signal to the SHG signal is proportional to the product of *ηα*_3_ *I*_00_ where *I*_00_ is the laser pulse peak irradiance and η is the PL efficiency = 26%.^1^

To precisely determine the excitation laser peak irradiance, the perovskite sample was removed and a glass substrate was placed at the focal point of the objective lens. After transmitting through the glass substrate, the average power (*P*_ave_) of the laser beam was measured by a power meter (Optical Power Meter 1917‐R, Newport). By considering the laser pulses with Gaussian profiles for both its temporal and its spatial domain, the laser pulse peak irradiance was calculated by *I*_00_ = 2(1−*R*)*P*_ave_/(*f*_RR_τ_p_π^3/2^ω^2^), where *R* is the Fresnel reflectance of the interface between the perovskite and substrate, *f*_RR_ is the pulse repetition rate, τ_p_ is the pulse duration (HWe^−1^M), and ω is the minimum beam waist and was measured to be 3 µm by the laser‐induced damage measurements as described in the following.

To investigate both the photostability and the laser‐induced damage, the 3PPL was measured as a function of exposure time to the laser pulses at a peak irradiance of 52 GW cm^−2^. No observable change was found within 5 min exposure times (or 3 × 10^8^ laser pulses). All the measurements presented here were obtained within 5 min exposure times. As the exposure time was extended to 1 h, however, an observable decrease in the 3PPL was detected, see an example in Figure S8 (Supporting Information). After the 1 h exposure time, a dark spot was visible, which is attributed to laser‐induced damage on the sample.

For excitation wavelength dependence, 3PPL measurements were conducted using the following apparatus. The excitation laser pulses (repetition rate: 1 kHz, wavelength range: 900–1200 nm, pulse width: 2τ_p_ ≈ 150 fs) were generated by an optical parametric amplifier (TOPAS‐C, Light‐Conversion) pumped by a regenerative amplified femtosecond Ti:sapphire laser system (wavelength: 800 nm, repetition rate: 1 kHz, pulse energy: 3 mJ, pulse width: 2τ_p_ ≈ 150 fs, Libra, Coherent), which was seeded by a femtosecond Ti:sapphire oscillator (repetition rate: 80 MHz, pulse width: 2τ_p_ ≈ 100 fs, wavelength: 800 nm, Vitesse TM 800‐2, Coherent). The laser pulses were focused by a lens onto the sample with a spot diameter of 2ω ≈ 10 µm. The 3PPL at an angle of 3° to the reflected direction was filtered by a short‐pass filter and was recorded with the spectrometer (QEpro, Ocean Optics). The average power of the laser pulses was measured by the optical power meter (Optical Power Meter 1917‐R, Newport) with the appropriate sensor (Detector 919P‐003‐10, Newport). More details about this setup can be found elsewhere.^29^

## Conflict of Interest

The authors declare no conflict of interest.

## Supporting information

Supplementary
