# Supplementary material for: Layered Hybrid Perovskites for Highly Efficient Three‐Photon Absorbers: Theory and Experimental Observation
Source: Adv Sci (Weinh). 2018 Dec 20;6(4):1801626. doi: 10.1002/advs.201801626 (PMC6382301; doi:10.1002/advs.201801626)
Supplement: Supplementary file 1 — Supplementary [file ADVS-6-1801626-s001.pdf]

Copyright WILEY-VCH Verlag GmbH & Co. KGaA, 69469 Weinheim, Germany, 2018.

ADVANCED SCIENCE
Open Access

Supporting Information

for *Adv. Sci.,* DOI: 10.1002/advs.201801626

Layered Hybrid Perovskites for Highly Efficient Three-Photon Absorbers: Theory and Experimental Observation

*Shunbin Lu, Feng Zhou, Qi Zhang, Goki Eda, and Wei Ji**

Supporting Information

Layered hybrid perovskites for highly-efficient three-photon absorbers: Theory and experimental observation

SHUNBIN LU,[1,2,*] FENG ZHOU,[2,*] QI ZHANG,[2] GOKI EDA,[2,3,4] AND WEI JI [2,1,**]

[1]SZU-NUS Collaborative Innovation Centre for Optoelectronic Science & Technology, International Collaborative Laboratory of 2D Materials for Optoelectronic Science and Technology of Ministry of Education, College of Optoelectronic Engineering, Shenzhen University, Shenzhen, Guangdong 518060, P. R. China

[2]Department of Physics, National University of Singapore, Singapore 117551, Singapore

[3]Department of Chemistry, National University of Singapore, Singapore 117542, Singapore

[4]Centre for Advanced 2D Materials, National University of Singapore, Science Drive 2, Singapore 117546, Singapore

*Both authors make equal contribution.

**Email address: phyjiwei@nus.edu.sg

Table S1 Parameters used in the calculation of 3PA coefficients

Parameters	$(C_4H_9NH_3)_2PbBr_4$
Active atom density, N	2×10^{21} cm^{-3} *
Refractive index, n_0	2.3 **
2D Bandgap, E_g	3.4 eV ***
2D Binding Energy, E_b	0.33 eV ***
2D Effective Mass, m^*_{2D}	0.17 m_e ****
3D Effective Mass, m^*_{3D}	0.25 m_e *****
2D Effective Bohr Radius, a_B^{2D}	16.4 Å ***
3D Effective Bohr Radius, a_B^{3D}	11.2 Å *****
2D Exciton Energy, $E_{1s} = E_g - E_b$	3.07 eV ***
$\Gamma_{1s} = \Gamma_{2p} = \Gamma_{3p}$	0.18 eV ***

Note: * refers to Ref. [S1]; ** refers to Ref. [S2]; * from this work; **** refers to Ref. [S3] and ***** refers to Ref. [S4].**

Table S2 Crystal data and structure refinement for $(C_4H_9NH_3)_2PbBr_4$

Empirical formula	C_8 H_{24} Br_4 N_2 Pb
Formula weight	675.12
Temperature	293(2) K
Wavelength	0.71073 Å
Crystal system	Orthorhombic
Space group	Cmca

Unit cell dimensions *	a = 27.548(2) Å α = 90°.<br>b = 8.3249(7) Å β = 90°.<br>c = 8.2064(7) Å γ = 90°.
Volume	1882.0(3) $Å^3$
Z	4
Density (calculated)	2.383 Mg/m^3
Absorption coefficient	17.438 mm^{-1}
F(000)	1232
Crystal size	0.201 × 0.146 × 0.058 mm^3
Theta range for data collection	2.958 to 28.261°.
Index ranges	-36 ≤ h ≤ 36, -11 ≤ k ≤ 11, -8 ≤ l ≤ 10
Reflections collected	9514
Independent reflections	1190 [R_{int}= 0.0678]
Completeness to theta = 25.242°	99.8 %
Absorption correction	Semi-empirical from equivalents
Max. and min. transmission	0.7457 and 0.4498
Refinement method	Full-matrix least-squares on F^2
Data / restraints / parameters	1190 / 5 / 39
Goodness-of-fit on F^2	1.110
Final R indices [I>2sigma(I)]	R_1 = 0.0655, wR_2 = 0.1534
R indices (all data)	R_1 = 0.0897, wR_2 = 0.1673
Extinction coefficient	n/a
Largest diff. peak and hole	3.996 and -4.241 e $Å^{-3}$

*Note: each unit has two layers of $PbBr_4^{-2}$

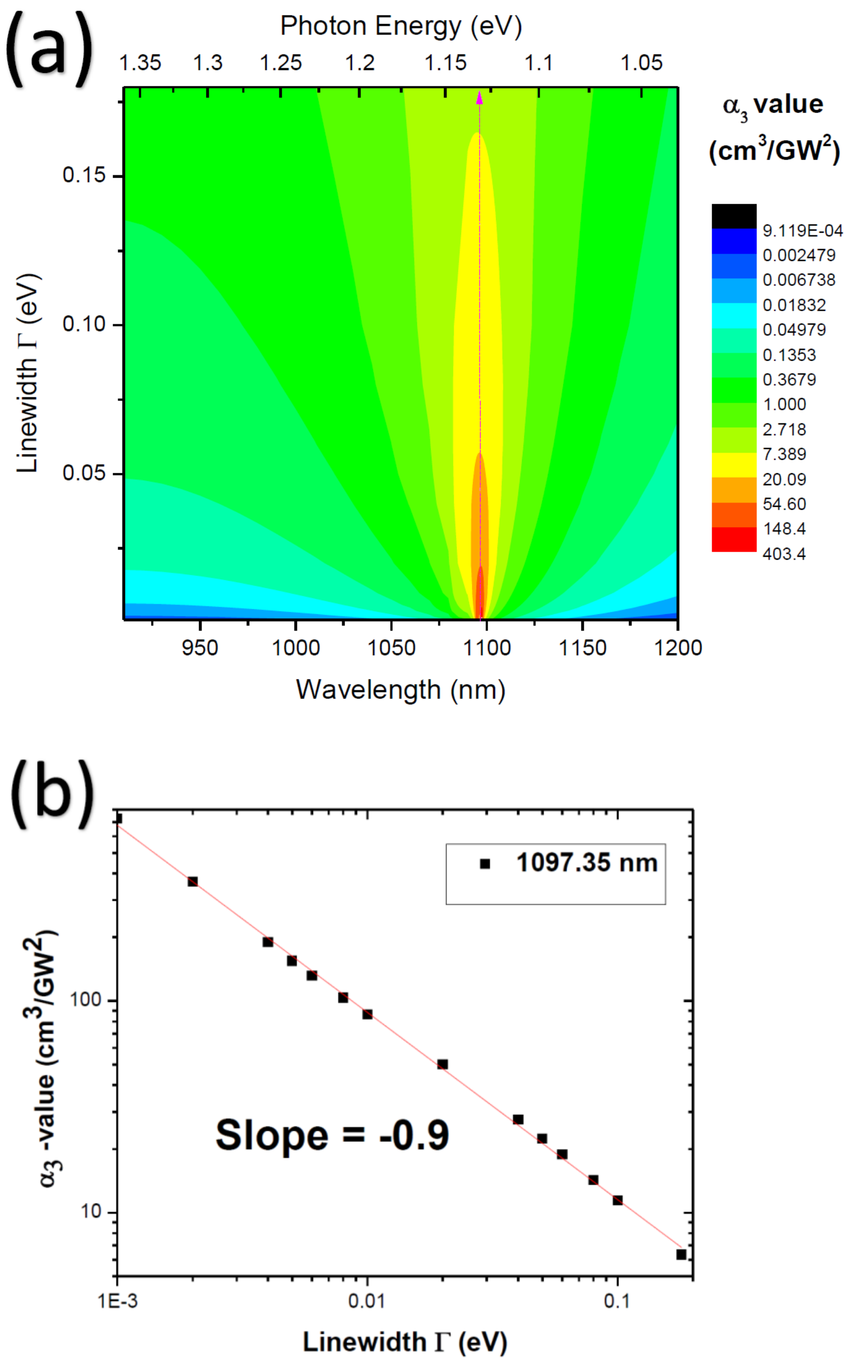

Fig. S1 Numerical modeling of (**a**) 3PA coefficient spectra and (**b**) maximal value as a function of the linewidth, Γ_f.

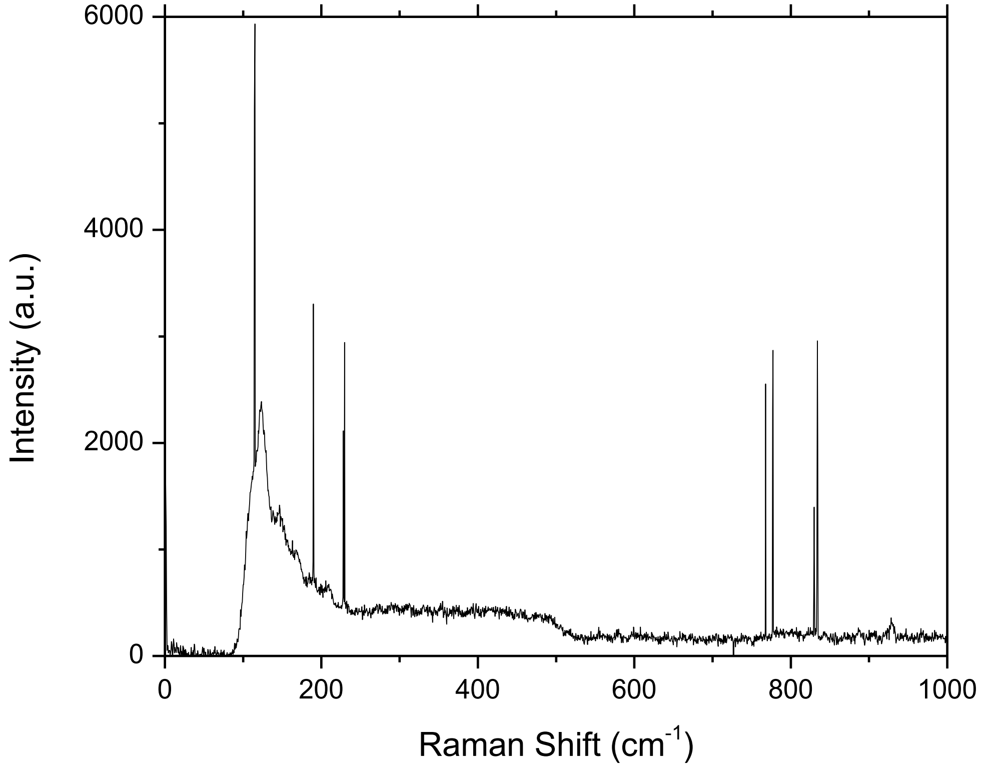

Fig. S2 Raman spectrum of a $(C_4H_9NH_3)_2PbBr_4$ flake excited by 532-nm laser beam, indicating a broaden band peaked at around 123 cm^{-1}.

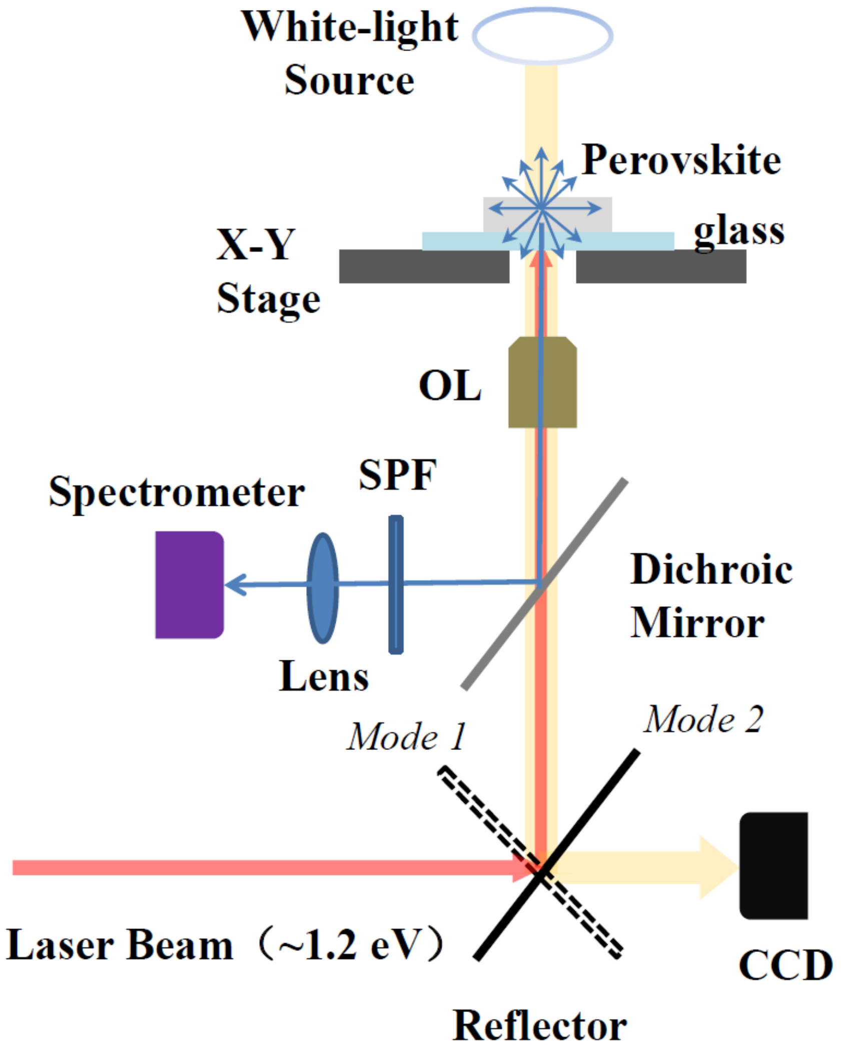

Fig. S3 A schematic for the microscopic setup (not up to the scale). **Laser**: A CW He-Cd laser (Wavelength: 325 nm) or a Light Conversion Pharos-9W fs oscillator with wavelength: 1030 nm, repetition rate: 1 MHz, and pulse duration: $FWe^{-1}H$ ~220 fs; Objective Lens **(OL):** Nikon Plan Fluor 20X/0.50, WD 2.1 mm; **Dichroic Mirror:** Di02-R635-Semrock-25 × 36; Short-Pass Filter **(SPF):** FGS900M Throlab 315-710 nm; **Spectrometer:** Model Ocean Optics QEpro; and **CCD:** Model TOUPCAM-UCMOS03100KPA.

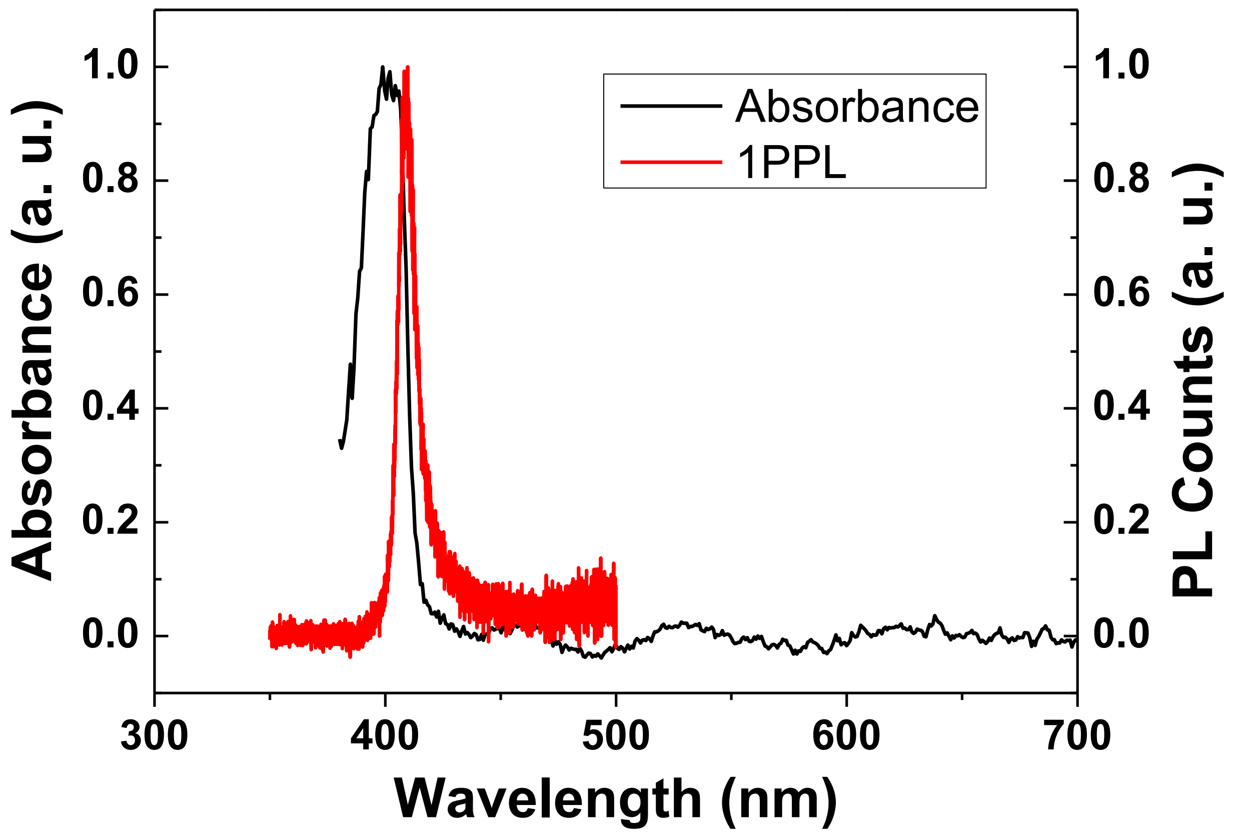

Fig. S4 Measured spectra for both one-photon absorption and one-photon-excited photoluminescence (1PPL). Thickness of layered perovskite $(C_4H_9NH_3)_2PbBr_4$: 140 nm.

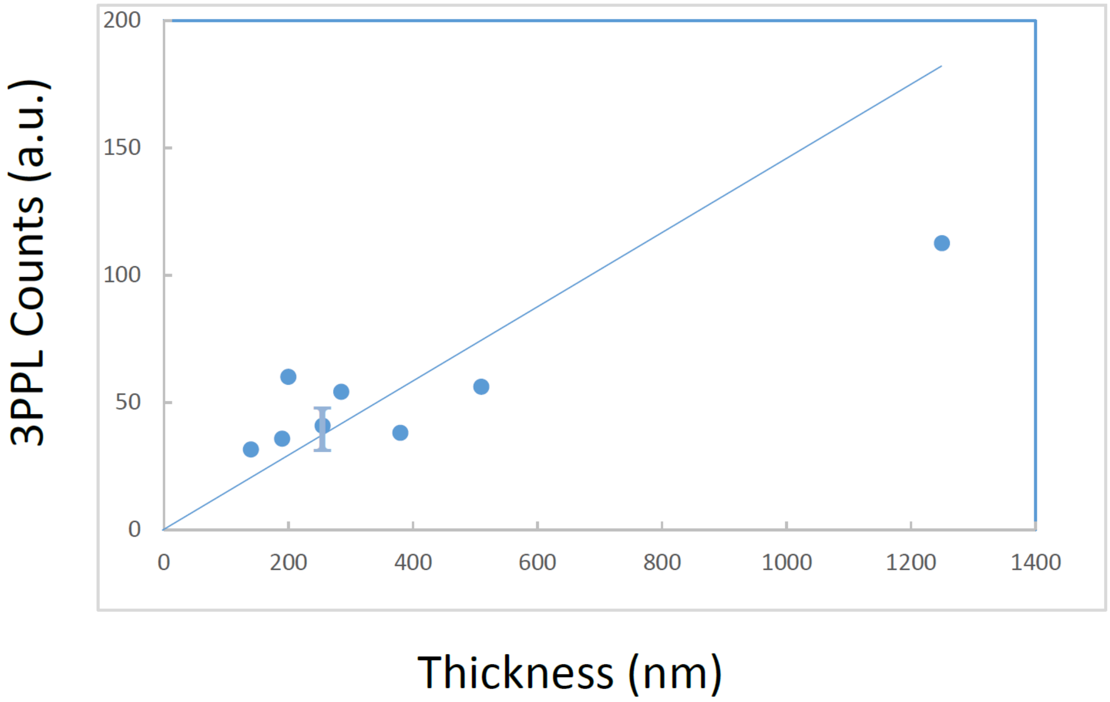

Fig. S5 Thickness-dependent 3PPL measured at a peak irradiance of 45 GW/cm^2 for layered perovskite $(C_4H_9NH_3)_2PbBr_4$. The line is a guideline for the eye.

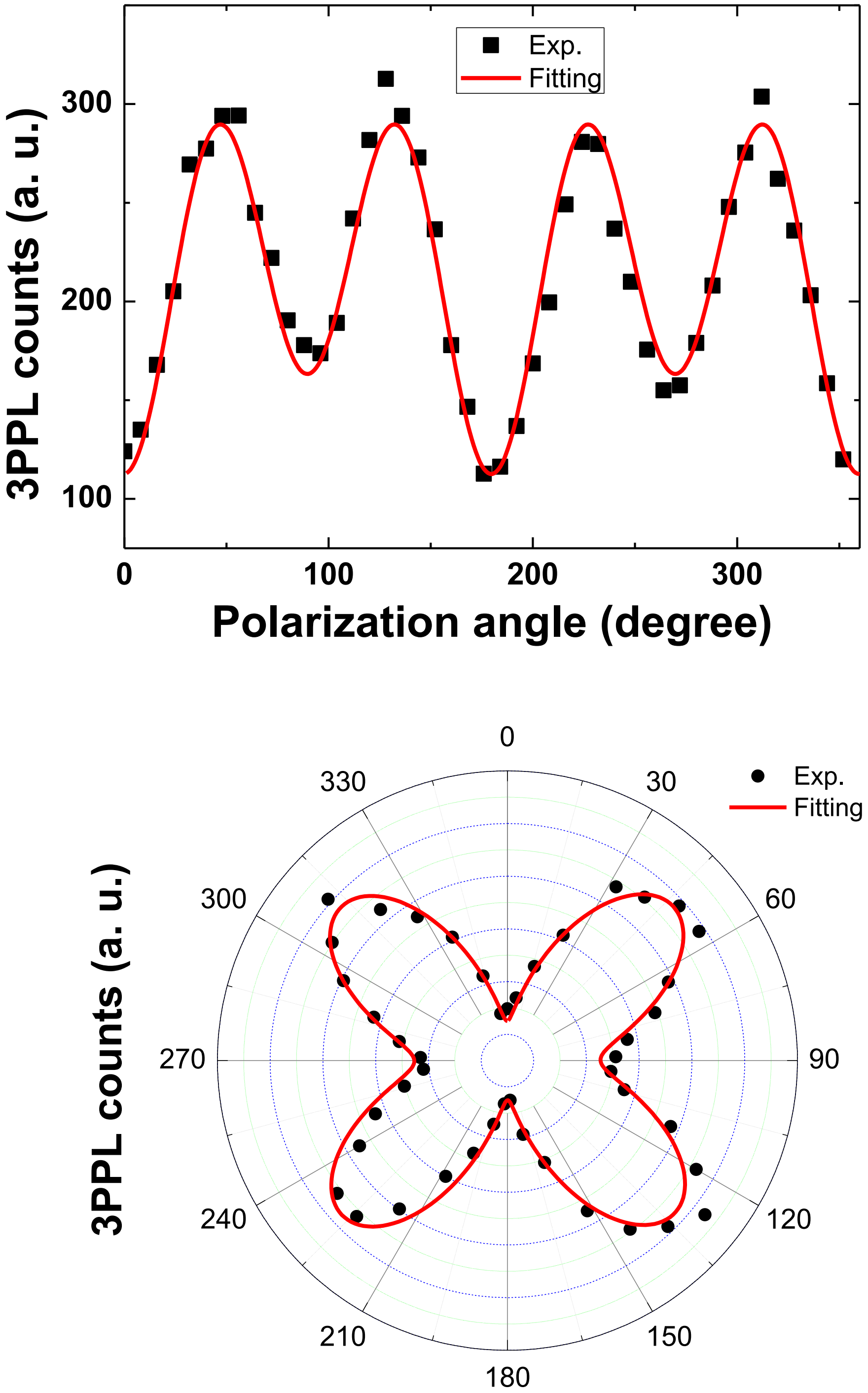

Fig. S6 Polarization angle dependence of 3PA at an excitation wavelength of λ_{ex} = 1030 nm along the [1 0 0] direction for layered perovskite $(C_4H_9NH_3)_2PbBr_4$.

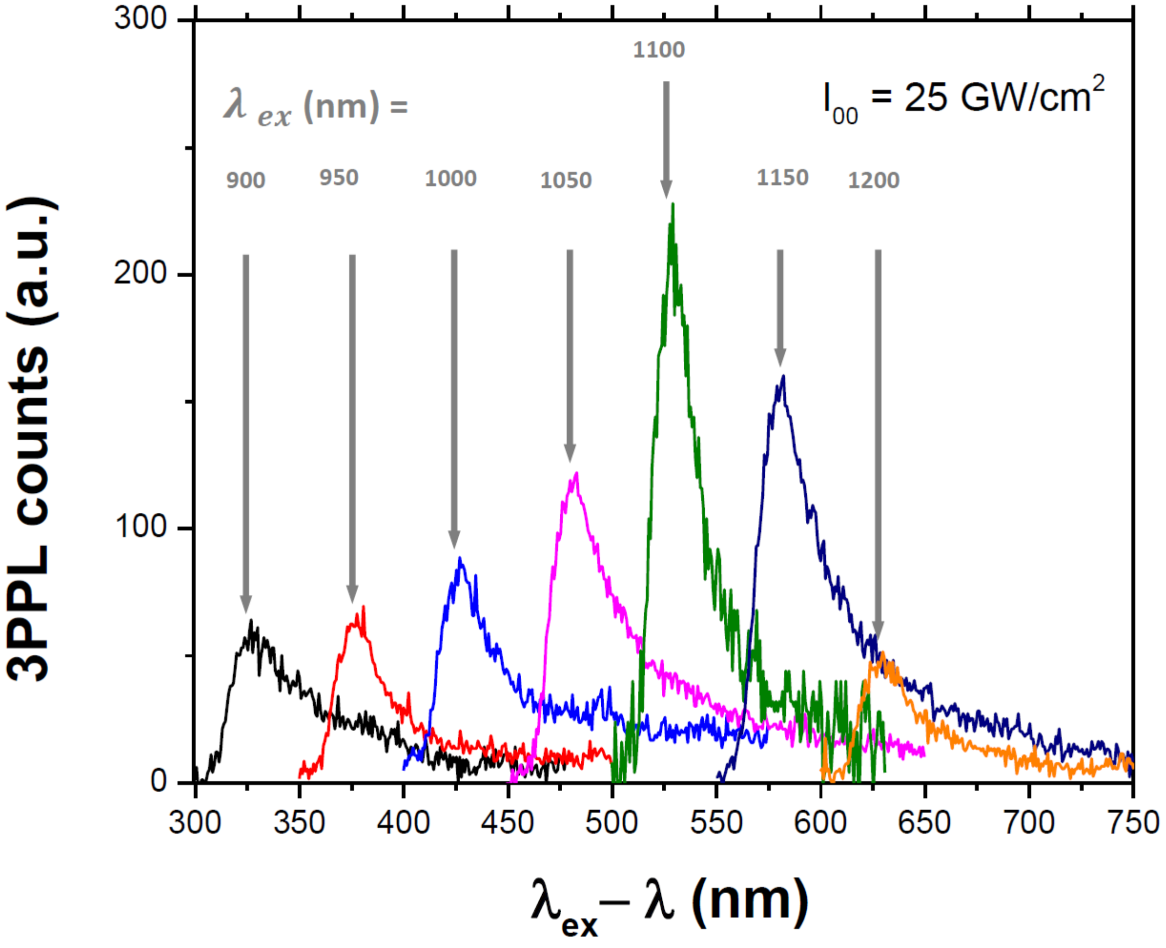

Fig. S7 Wavelength dependence of 3PPL obtained at a peak irradiance of 25 GW/cm^2 from layered perovskite $(C_4H_9NH_3)_2PbBr_4$.

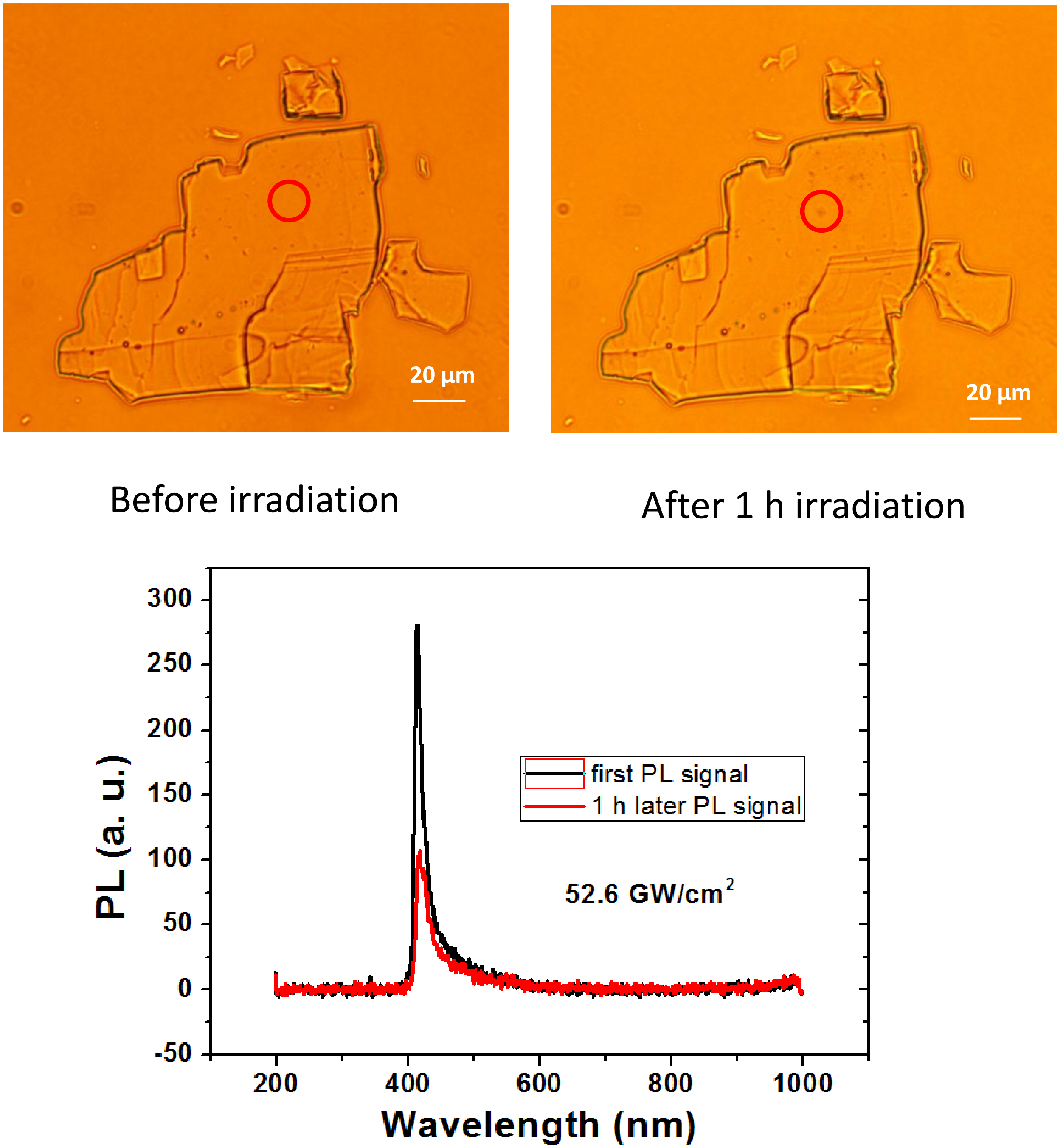

Fig. S8 Photo-stability measurements. The CCD photo (top left) shows the red-circled area of the sample before exposure to the femtosecond laser pulses. The CCD photo (top right) shows the same area of the sample after one-hour exposure time (wavelength: 1030 nm, repetition rate: 1 MHz, and peak irradiance: 52.6 GW/cm^2). The bottom spectra show the difference in the 3PPL measured at the first minute and 60 minutes of the excitation. A dark spot located at the center of the red circle is visible after the one-hour laser irradiation.

References

S1. F. O. Saouma, C. C. Stoumpos, J. Wong, M. G. Kanatzidis, J. I. Jang, Nature Comm. **2017**, 8, 742.

S2. I. Abdelwahab, G. Grinblat, K. Leng, Y. Li, X. Chi, A. Rusydi, S. A. Maier, K. P. Loh, ACS Nano **2017**, 12, 644-650.

S3. K. Tanaka, T. Takahashi, T. Kondo, K. Umeda1, K. Ema1, T. Umebayashi, K. Asai, K. Uchida, N. Miura, Japanese Journal of Applied Physics **2005**, 44, 5923.

S4. J. H. Lei, Y. Q. Zhao, Q. Tang, J. G. Lin, M. Q. Cai, Physical Chem. Chemical Phys. **2018**, 20, 13241-13248.
